# Point-of-care biosensors for infectious disease diagnosis: recent updates and prospects

**DOI:** 10.1039/d5ra03897a

**Published:** 2025-08-19

**Authors:** Mansi Parihar, Niharika W. N., Sahana, Rajib Biswas, Budheswar Dehury, Nirmal Mazumder

**Affiliations:** a Department of Bioinformatics, Manipal School of Life Sciences, Manipal Academy of Higher Education Manipal 576104 India budheswar.dehury@manipal.edu; b Department of Physics, Tezpur University Assam India; c Department of Biophysics, Manipal School of Life Sciences, Manipal Academy of Higher Education Manipal Karnataka 576104 India nirmal.mazumder@manipal.edu

## Abstract

The ongoing demand for rapid, accurate, accessible diagnostics has significantly increased point-of-care (POC) biosensors. This review provides an overview of diverse biosensors, focusing on their principles, components, detection mechanisms, and applications in infectious disease diagnosis. We explore how these biosensors utilize various transduction techniques-such as current modulation, refractive index shifts, and mechanical resonance to convert biorecognition events into measurable signals. The importance of biosensors in detecting infectious diseases such as COVID-19, HIV, Tuberculosis, and Malaria is highlighted, particularly for early detection in resource-limited settings. However, persistent challenges remain in achieving integrated, miniaturized platforms capable of real-time, multianalyte detection. Additionally, the full potential of biosensors is yet to be realized owing to limited clinical translation, scalability issues, and insufficient integration with digital health technologies. This review identifies these critical areas for future innovation and discusses strategies to increase diagnostic accuracy, accessibility, and global health impact.

## Abstract 2

The ongoing demand for rapid, accurate, accessible diagnostics has significantly increased point-of-care (POC) biosensors.

## Graphical abstract

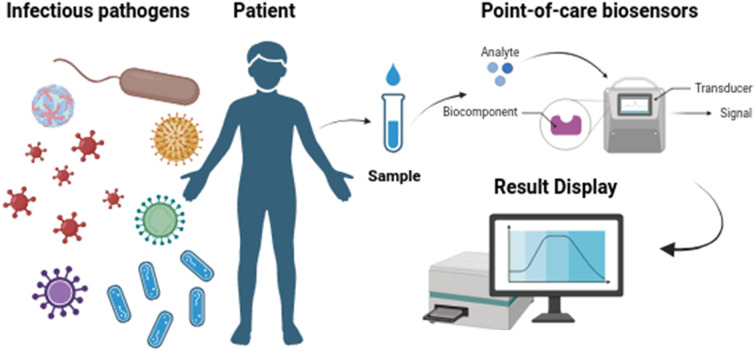

## 1. Introduction

In the evaluation and advancement of global health, access to appropriate diagnostic tools is crucial. Traditional pathogen detection techniques, such as culturing, enzyme-linked immunosorbent assay (ELISA), or polymerase chain reaction (PCR), often require advanced infrastructure and well-equipped laboratories. These techniques may require highly trained clinicians, expensive instruments and reagents or complex assay protocols.^1^ Many such techniques require multiple patient visits to health centers.^2^ These limitations hinder the flexibility of traditional methods for timely and accurate diagnosis and treatment.^1^ For example, the COVID-19 pandemic emphasized the urgency for accurate, timely and affordable diagnostic tools for healthcare management.^2^

To overcome these limitations, the development of point-of-care (POC) methods for pathogen detection is attracting increasing interest. POCT is a form of clinical laboratory testing conducted near the site where patients receive care. This allows for immediate results to be generated and sent to doctors for clinical decision-making. The standards for a POC test are summarized by the acronym REASSURED, that is further discussed in the following section. This means that POC tests should ideally have real-time connectivity, ease of sample collection, affordability, sensitivity, specificity, and user-friendliness, be rapid and robust, be equipment free, and be delivered to the end user. The demand for POC testing devices from healthcare authorities, professionals and the public is increasing exponentially. This is highly relevant in environments where infrastructure is limited. The immediate detection of infectious agents is important in early diagnosis and treatment. The implementation of rapid, precise and dependable POC devices during the early stages of outbreaks in endemic regions could significantly enhance diagnostic capabilities and clinical management.^2^

Among the noteworthy bioanalytical techniques for quick and precise detection in biological fluids, biosensor-based approaches are prominent. Biosensors are analytical devices that consist of a biological recognition element, a transducer and a signal processor (reader). They convert biochemical signals into measurable outputs such as electrical or optical signals.^1^ This review focuses on recent advancements in electrochemical, optical, and piezoelectric biosensors for infectious disease diagnosis in the context of POC applications. Electrochemical biosensors have been developed and applied for infectious disease diagnostics because of their high sensitivity, low cost, simplicity, dependability, quick response, miniaturization, durability and POC compatibility.^1^ Optical biosensors are used because of their high accuracy and potential to provide rapid health monitoring and noninvasive disease diagnosis, and piezoelectric biosensors, because of their importance as reliable POC infectious disease diagnostic tools, will also be explored.^3,4^

Considering the growing demand for faster and more accessible diagnostics, this review delves into how recent innovations in POC biosensors are being put into action. Rather than revisiting the technical classifications, we focus on how these tools make a difference from speeding up disease detection to expanding diagnostic reach in resource-limited settings. In addition to their real-world applications, we discuss the practical hurdles these systems still face and where future improvements are headed. The goal is to highlight that biosensors are no longer limited to research labs; they are beginning to have a real impact on real-world healthcare.

## 2. Types of biosensors

### 2.1. Electrochemical biosensors

**Fig. 1:**
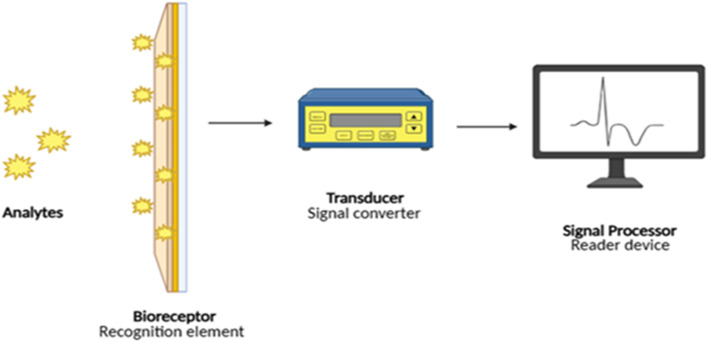
Workflow of an electrochemical biosensor.

An electrochemical biosensor is a device used for detecting specific analytes present in biological samples. They combine a physicochemical transducer and a biological recognition element for detection.^5^ These devices can operate independently without any support from other devices. To measure analytes precisely and sensitively, chemical reactions are converted into electrochemical signals such as current and voltage.^6^ The components of an electrochemical biosensor device include a biological recognition element that acts as a biological receptor/bioreceptor, an electrochemical transducer that acts as a signal transducer/detector element, and a signal processor that acts as a detector circuit/reader device. Biorecognition elements are those elements that act as biological receptors for detecting and binding to specific analytes from a sample. The biorecognition element uses an immobilization technique to attach to the transducer. The performance of a biosensor is highly influenced by the immobilization technique. Common immobilization techniques include physical adsorption, covalent bonding (gold–thiol interactions where thiol-modified aptamers are commonly immobilized on gold electrodes), the immobilization of antibodies covalently on gold surfaces (photochemical immobilization technique), the use of graphene surfaces modified by polymers, the entrapment method, the use of polypyrrole films (electropolymerization), *etc.*^7,8^ Traditional examples consist of antibodies, enzymes, and whole cells, whereas the latter examples consist of aptamers and peptides, which provide enhanced stability, versatility, and flexibility.^3^ Common examples include glucose oxidase (for glucose), which can be represented by glucose oxidase (GOx): O_2_ + glucose GOx → H_2_O_2_ + gluconic acid; oxidase enzymes (for H_2_O_2_), which can be expressed as follows: H_2_O_2_ + donor HRP → 2H_2_O + oxidized donor, lactate oxidase, polyamine oxidase, and urease nanoparticles.^4^ Natural receptors can be unstable, and artificial receptors such as molecularly imprinted polymers (MIPs) and surface imprinted polymers (SIPs) offer portability and selective binding through covalent, semicovalent and noncovalent interactions, including hydrogen bonds, hydrophobic interactions, electrostatic interactions and metal chelation. These novel artificial receptors are synthesized in 3 steps: the assembly of functional monomers and templates, polymerization and template removal.^9^ The electrochemical transducer converts biological interactions into electrical signals.^6^ It works through reference, counter and working electrodes that measure electrochemical changes.^1^ Electrodes are key for electron and bioagent flow,^10^ and modifying their surface improves their sensitivity and selectivity.^11^ Nanomaterials are crucial for sensitivity and specificity. Some examples include gold nanostructures (3D gold nano/microislands (NMIs) and gold nanoparticles (AuNPs)), whose increased active surface area leads to a significant increase in the performance of the biosensor; graphene and carbon nanomaterials (as they have unique physical structures and chemical and electrical properties); carbon nanostructure–polymer composites; carbon nanotubes (used for label-free detection of small molecules); and metal oxide–ZnO nanostructures (used as a surface layer owing to their high isoelectric point and strong binding affinity toward biomolecules).^8,12^ The transducer enables detection *via* current, potential or impedance, making the biosensor an affordable, quick POC device.^12^ The signal processor converts raw electrical signals from the transducer into readable data, facilitating analyte quantification and assessing the detection limit and reliability. It can be built within the device rather than as a separate component. The signal is acquired by electrochemical measurement devices such as potentiostats *via* electrochemical reaction mechanism techniques such as differential pulse voltammetry (DPV), cyclic voltammetry (CV), potentiometry, conductance, and electrochemical impedance spectroscopy (EIS).^12–15^ Data processing involves quantification *via* calibration curves, signal normalization, and calculation of key metrics such as the limit of detection (LOD), sensitivity, selectivity, repeatability, and stability. The LOD formula is expressed as LOD = 3*σ*/*S* (where *σ* is the standard deviation of the blank signal and *S* is the sensitivity).^13^ Statistical validation ensures precision and specificity for clinical applications.^16^ Machine learning (ML) enhances data handling, anomaly detection, and sensor performance among interferences for advanced analysis.^4^ Signal amplification techniques support affordable, portable POC devices such as the READ system (rapid electroanalytical device), enabling rapid results outside traditional labs.^5,16^ The three core components—the recognition element, transducer, and processor – work hand-in-hand to capture a biological event, transform it into an electrical signal, and then interpret that signal into meaningful diagnostic information to provide the user (Fig. 1).

The fundamental working principle of electrochemical biosensors is to convert a biological or biochemical event into a measurable and identifiable electrical signal.^17^ When an alternating current (AC) or voltage is applied, these biosensors detect changes in the reactive and resistive properties of the electrode surface, which leads to the generation of an electrical signal corresponding to the biological interaction.^18^ In this device, a biological recognition event, such as the binding of an analyte to an enzyme, antibody, or nucleic acid, is transduced into an electrical signal in the form of voltage, current, or impedance, depending on the sensor design.^4^ Properties such as molecular recognition, specificity and signal transduction efficiency contribute to the process of signal transduction. The device uses working, reference, and counter electrodes as key components for the transfer of electrons. They also convert biological interactions into readable signals. To read and understand these signals and interpret the analytical data, multiple techniques, such as cyclic voltammetry, differential pulse voltammetry, electrochemical impedance spectroscopy and chronocoulometry, are used.^3^ Chronocoulometry is used in the case of aptamers to calculate the density of the aptamers immobilized on the surface. The equation is represented by*Γ*_DNA_ = *Γ*_0_·(*z*/*m*)·*N*_A_,where *Γ*_0_ represents the amount of redox marker confined near the electrode surface. *Γ*_DNA_ is the probe surface density in molecules per cm^2^, *m* is the number of bases in the probe DNA, *z* is the charge of the redox molecule, and *N*_A_ is Avogadro's number.^13^

### 2.2. Optical biosensors

Optical biosensors have gained global attention because of their immediate and sensitive detection of biomarkers, resulting in less background interference, especially in healthcare and clinical settings.^19,20^ The performance of these methods is further enhanced by their resistance to electromagnetic interference and low noise levels, which helps ensure accurate results in complex diagnostic environments.^21^ Unlike electrochemical biosensors, optical biosensors rely on photonic signal transduction mechanisms, where various interactions between the incident light and the chemically modified sensor surfaces, such as refractive index shifts, absorbance, scattering, and reflectance, are detected directly without the dependence on electronic conductivity.

**Fig. 2:**
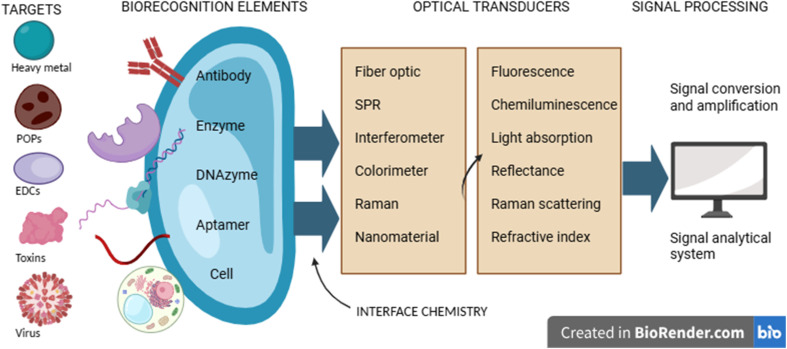
Schematic representation of the optical biosensing process.^24^

The fundamental step is surface functionalization of the sensor surfaces. The biorecognition elements, such as antibodies, nucleic acids, or aptamers, are immobilized onto the sensor surfaces by chemically modifying them to ensure the controlled orientation, density, and stability of the biomolecules. For gold-coated surfaces, thiol–gold self-assembled monolayers (SAMs) are typically used. These SAMs form through strong Au–S covalent bonds, creating stable and densely packed layers that support the uniform attachment of bioreceptors. Alternatively, glass and silica surfaces usually undergo silanization reactions where alkoxysilanes hydrolyze and condense to form siloxane linkages, allowing covalent functionalization with amine, carboxyl, or epoxy groups. Carbodiimide-mediated 1-ethyl-3-(3-dimethylaminopropyl)carbodiimide/*N*-hydroxysuccinimide (EDC/NHS) coupling is widely used to covalently link carboxylated surfaces to primary amines in proteins or aptamers, resulting in stable amide bond formation.^22,23^ These immobilization strategies critically determine sensor sensitivity and specificity by influencing receptor accessibility and signal transduction efficiency (Fig. 2).

The optical biosensor works *via* the components: a biorecognition element, a target analyte, an optical transducer, and a signal processor. First, the sensor surface is chemically modified to immobilize specific biorecognition elements. The biorecognition element will bind or react with the target analyte present in the sample *via* mechanisms such as antigen–antibody interactions or enzyme–substrate reactions.^25^ This biological interaction causes some changes in optical properties, such as a shift in fluorescence, absorbance, reflectance, or the refractive index. The produced change is then detected by the optical transducer as an optical signal, which is later read by the signal processor.^19^ This signal is analyzed to identify and establish the concentration of the analyte present in the sample.^26^

Optical biosensors are able to provide comprehensive information on analyte concentration and binding kinetics. They depend on various optical principles and mechanisms to perform precise quantitative bioassays.^23^ During detection, the incident light interacts with the functionalized surface to produce quantifiable optical changes. For example, in absorption-based optical biosensors, the analyte absorbs specific wavelengths of light, which changes their characteristics. The reduction in transmitted light intensity is measured. It forms the basis of the absorbance-based quantification, described by the Beer–Lambert law. The absorbance (*A*) was calculated *via* the following formula:
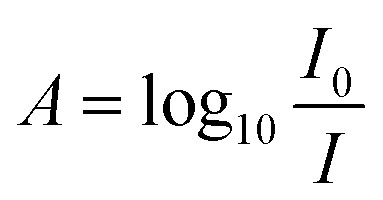
where *I*_0_ is the intensity of the incident light and where *I* is the intensity of the transmitted light. The absorbance correlates with the analyte concentration, and the relationship is given by:*A* = *ε* × *l* × *C*where *ε* is the molar absorptivity (L mol^−1^ cm^−1^), *l* is the optical path length (cm), and *C* is the analyte concentration (mol L^−1^).

In more advanced systems such as surface plasmon resonance (SPR), analyte binding causes the refractive index to change near a metal surface (typically gold or silver), which in turn shifts the resonance wavelength or angle of propagating surface plasmons. The sensor's sensitivity (*S*) is defined as:
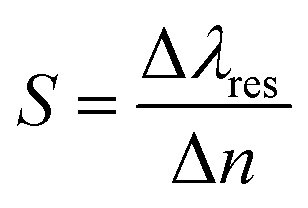
where Δ*λ*_res_ is the shift in the resonance wavelength and where Δ*n* is the change in the refractive index caused by analyte binding. This shift is detected as a measurable optical signal without the need for chemical labels.

Advancements in the field of optical biosensor technology over the past two decades have led to the development of several sophisticated platforms. These methods include optical fiber plasmonic coatings,^27,28^ photonic crystal waveguide cavity resonators,^29^ surface plasmon resonance (SPR) systems,^30^ localized surface plasmon resonance (LSPR),^31,32^ photonic crystal fibers,^33^ and metasurface-based sensors.^34,35^ These photonic crystal fibers, metasurfaces, and nanoplasmonic coatings help the sensors interact better with light and are made *via* techniques such as chemical etching, vapor deposition, or self-assembly. These technologies allow for real-time, label-free detection by tracking the changes in the refractive index, eliminating the requirement of complex tags or labels. Compared with magnetic sensors, optical biosensors offer better sensitivity, making them more suitable for clinical diagnostics.^36^ Depending on whether an external label or tag is needed, optical biosensors can operate in two primary detection modes: label-free and label-based detection. The label-based detection mode uses radioactive or fluorescent labels for the detection of analytes, which produces a detectable signal upon binding with the target molecule. These methods are sensitive and highly specific to analytes and are useful in applications such as immunoassays and DNA microarrays.^37^ However, label-free biosensors do not require any enzyme tags or labels. Rather, they detect alterations directly from the analyte–sensor surface interaction. Refractive index shifts, mass accumulation, or structural alterations are a few examples of these alterations.^38^

Optical biosensors stand out for their high sensitivity, specificity, and ability to detect biomarkers in real-time without the use of labels. They have been shown to be flexible and effective in various techniques. However, their cost and technical complexity can be a hurdle, especially for their use outside the laboratory. These systems need to be simpler, straightforward, and user-friendly so that they can significantly increase their value in everyday diagnostic settings.

### 2.3. Piezoelectric biosensors

Unlike optical biosensors that measure analytes *via* changes in light or refractive indices, piezoelectric biosensors operate by measuring mechanical changes and resonant frequency shifts. These sensors, commonly called mass-to-frequency converters, use the piezoelectric effect to convert mechanical inputs such as strain, pressure, or motion into equivalent electric outputs. A piezoelectric material, commonly quartz, is coated with selective biorecognition agents such as enzymes, antibodies, or living cells. When the target analyte attaches to the biorecognition element on the sensor surface, it slightly increases the overall mass, disturbing the mechanical balance. This alteration modifies the natural vibration frequency of the piezoelectric crystal. The change in frequency is subsequently converted into an electrical signal, indicating the detection of the analyte.^39,40^ These biosensors operate by monitoring surface acoustic waves, which respond sensitively to even small changes in mass. The frequency change is proportional to the mass change, a factor explained by the Sauerbrey equation:
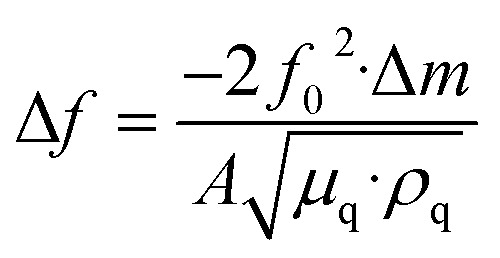


In the Sauerbrey equation, Δ*f* represents the frequency shift in hertz (Hz), *f*_0_ is the fundamental frequency of the quartz crystal (Hz), Δ*m* denotes the change in mass on the crystal surface in grams (g), and *A* is the active surface area of the electrode in square centimeters (cm^2^). The material properties of the quartz crystal are characterized by *μ*_q_, the shear modulus of quartz, with a value of approximately 2.947 × 10^11^ g cm^−1^ s^−2^, and *ρ*_q_, the density of quartz, which is approximately 2.648 g cm^−3^.^41^ The above equation relies on the detection of frequency changes caused by mass binding on a quartz surface. However, most biosensors function in liquid environments, such as blood, urine, or buffer solutions. In these situations, viscous damping and fluid loading can lead to additional frequency shifts that the Sauerbrey model does not account for. The Kanazawa–Gordon equation addresses this limitation by estimating the frequency shift (Δ*f*) caused by the viscosity and density of the liquid in contact with the crystal.

Kanazawa–Gordon equation:
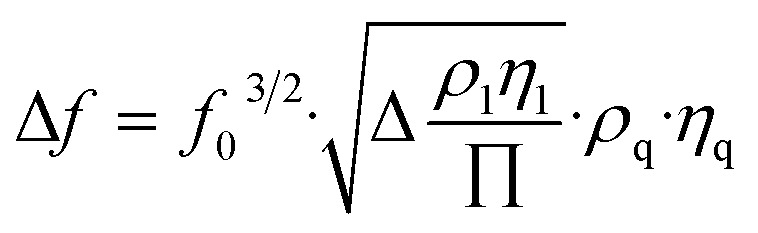


In the Kanazawa–Gordon equation, Δ*f* denotes the frequency shift (Hz), and *f*_0_ is the resonant frequency of the quartz crystal (Hz). *η*_l_ represents the dynamic viscosity of the liquid in pascal-seconds (Pa s), whereas *ρ*_l_ is the density of the liquid in kilograms per cubic meter (kg m^−3^). The quartz material is further described by *μ*_q_, the shear modulus of quartz, approximately 2.947 × 10^10^ N m^−2^, and *ρ*_q_, its density, approximately 2650 kg m^−3^. The constant π refers to the mathematical constant pi, with a value of approximately 3.1416.^42^ This equation is particularly critical when interpreting biosensing data in clinical samples, where liquid-phase interactions are predominant, such as in blood or saliva. This correction is crucial for clinical diagnostics, including the detection of viral pathogens such as SARS-CoV-2 *via* piezoelectric biosensors that function in physiological fluids.^13,43^ Piezoelectric transducers have been widely explored as chemical sensors *via* the same equation.^44^ However, the equation assumes ideal conditions, such as a rigid, thin film and a vacuum or air environment. To address this, modern biosensor designs often incorporate reference crystals (dual-crystal setups), where one crystal is used as an internal control to correct for environmental variations. More recent developments include integration with microfluidic systems and real-time data correction algorithms, which enhance sensitivity and reliability in complex biological samples. Modern sensor designs frequently utilize chemically stable coatings and dual-crystal references to overcome limitations, effectively compensating for nonspecific interactions and ambient interference.

#### 2.3.1. Materials for the piezoelectric assay

**Fig. 3:**
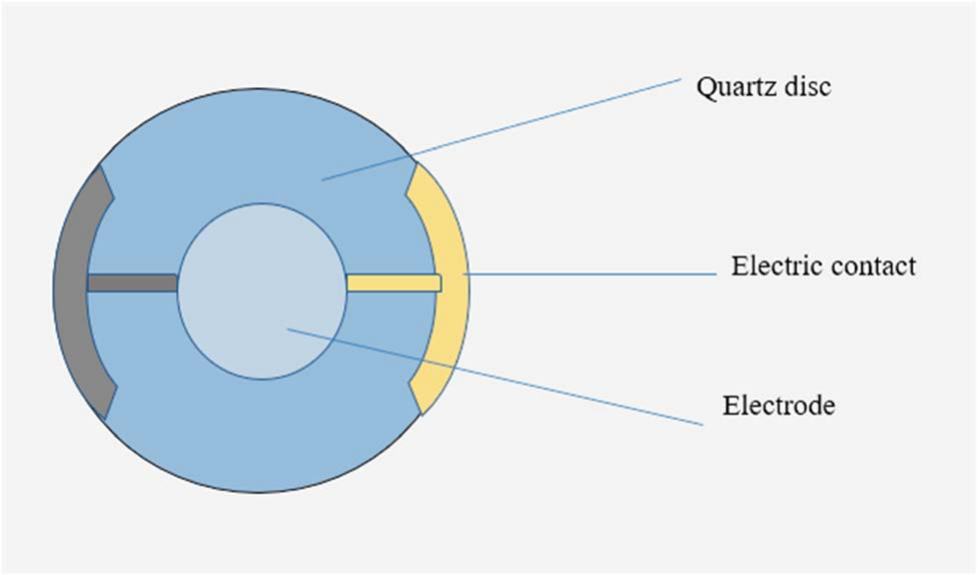
An example of a QCM sensor features a fundamental oscillation frequency of 10 MHz, a 20 mm diameter, and gold-coated electrodes.^46^

Piezoelectric biosensors utilize materials that transform mechanical stress into electrical signals, making them adaptable for various biomedical and analytical uses. Inorganic materials such as quartz, first discovered by the Curie brothers in 1880, along with Rochelle salt, BaTiO_3_, AlN, ZnO, and PZT, are appreciated for their stability and sensitivity.^45^ Synthetic polymers, including PVDF, polylactic acid, and polyamides, provide flexibility for compact, wearable sensors, with hybrid PVDF (polyvinylidene fluoride) films improving the overall performance. Materials of biological origin, such as Piezo1 ion channels, are increasingly favored for their natural compatibility with biological systems and their effectiveness in translating mechanical forces into cellular responses. Among available technologies, quartz crystal microbalances (QCMs) are the most commonly used owing to their low cost, ease of operation, and reliable sensitivity within the 1–20 MHz range. However, while more sensitive, devices that operate at higher frequencies are generally more fragile and less durable.^46^Fig. 3 presents a representative design of a QCM sensor used in piezoelectric biosensing. Recent advances have also introduced metal–organic frameworks (MOFs) as functional coatings to increase the sensitivity and surface area of piezoelectric biosensing platforms.^47^

**Fig. 4:**
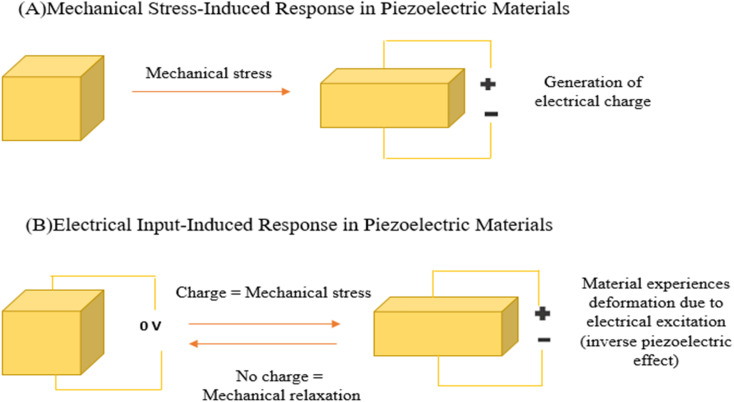
Illustration of how mechanical stress influences a piezoelectric material (A) and how the resulting electrical charge is generated in response to the applied stress (B).^46^

Piezoelectric biosensors depend on materials that respond to mechanical pressure by generating electrical signals. Scientists obtain these materials from diverse sources, including naturally occurring crystals, engineered polymers, and biologically derived substances. This variety allows them to develop sensors suited to specific healthcare and laboratory diagnostic needs. Piezoelectric biosensors use materials that produce electrical signals when subjected to mechanical stress, a phenomenon referred to as the piezoelectric effect. Typically, quartz crystals are modified with biomolecules that adhere to specific target analytes, changing the surface mass and causing a shift in the crystal's resonant frequency, as outlined by Sauerbrey's equation.^41,48^ In addition to mass detection, these sensors can react to mechanical forces such as bending or pressure. When a piezoelectric material is deformed, it polarizes intrinsic dipoles, creating an electric current and potential. When the force is released, a reverse current takes place. The magnitude of the signal is determined by the applied stress as well as the properties of the material, enabling the high sensitivity of biochemical detection and physiological monitoring (Fig. 4).

However, critical challenges remain, including sensor surface stability in complex biological fluids, difficulties in regenerating crystals, long incubation times, nonspecific adsorption of proteins or other biomolecules, and the loss of surface coatings during washing procedures.^49^ While still a subject of challenge, piezoelectric biosensors allow for rapid, extremely sensitive, and label-free detection and are thus ideally suited for rapid diagnostic applications. However, their susceptibility to physical and environmental stress degradation suggests the necessity of more resilient and reusable sensor configurations. These advancements highlight the effectiveness of piezoelectric biosensors in POC diagnostics, especially for detecting infectious diseases with high sensitivity and specificity. Piezoelectric biosensors are devices that detect interactions between biomolecules by converting changes in mass into frequency or voltage shifts. The core implementations include the Quartz Crystal Microbalance (QCM), Thickness Shear Mode (TSM), and Piezoelectric Quartz Crystal (PQC), which are all based on bulk acoustic wave (BAW) propagation. PQC is frequently used interchangeably with the QCM because of its shared operational principles. In contrast, surface acoustic wave (SAW) sensors function on the basis of wave propagation along the sensor surface. The various types of piezoelectric biosensors and their key features are summarized in Table 1.

**Table 1:** Classification and performance characteristics of biosensors for infectious disease detection

Type	Specification	Advantages	Disadvantages	References
**Electrochemical**				
Voltametric	Measures current while varying the potential over time also includes Differential pulse voltammetry (DPV) and Cyclic voltammetry (CV)	These systems offer high sensitivity, low cost, portability, and fast response with minimal sample use	Stable surface modifications and complex protocols are needed, and biological components may limit performance and need complex indicators	1
Amperometric	Measures current at constant potential, proportional to electroactive species concentration	Facilitates the measurement of analytes. Retains the general benefits of electrochemical biosensors	Shares general disadvantages: complexity in production, signal interference, and potential enzyme inhibition	1, 4 and 50
Potentiometric	Measures potential between electrodes at zero current, reflecting analyte concentration or activity	Good selectivity, sensitive, stable reference systems, low power consumption, noninvasive potential	Limited to ions and certain analytes, affected by ionic strength or matrix composition, production and calibration complexity	1, 4 and 5
Impedimetric	Measures impedance of electrode-solution interface (*e.g.*, Electrochemical impedance spectroscopy (EIS))	Label-free detection allows sensitive, real-time biomolecular analysis and is suitable for miniaturization and adaptable use	Temperature and matrix effects, complex interpretation, surface modification required and exposure to environmental noise	1, 18 and 51
Conductometric	Measures changes in conductivity near the electrode due to biochemical reaction	Easy setup, rapid feedback, wide detection range, ideal for small sample sizes and complex matrices	Requires signal amplification, limited specificity, temperature and pH dependent	1 and 5
Other types (Organic electrochemical transistor (OECT), photoelectrochemical, and electrochemiluminescent sensors)	Each uses specialized electrical or light-based detection principles	Enhanced sensitivity, integration with optical/electronic systems, suitable for multiplexed detection and portable formats	Integration and design complexity, high cost of specialized parts, need for advanced production	1

**Optical**				
Surface plasmon resonance (SPR)	Measures refractive index shifts at a metal–liquid interface due to biomolecular binding	High sensitivity, real-time monitoring, label-free, kinetic, and affinity analysis possible	Expensive instrumentation, limited to surface interactions, requires stable surface functionalization, sensitive to temperature and bulk refractive index changes	52
Ellipsometry	Measures changes in light polarization upon binding, precise surface analysis	Ultrasensitive to thin layers, label-free, suitable for surface binding studies	Requires clean, reflective surfaces, complex data interpretation	53
Absorbance/reflectance	Measures light absorbed/reflected due to analyte–enzyme/color interaction	Simple setup, cost-effective, compatible with basic lab equipment	Lower sensitivity, prone to interference, limited dynamic range	54
Scanning angle reflectometry (SAR)	Measures angle-dependent reflectance to analyze refractive index and layer thickness	High precision for layer thickness and surface concentration, label-free	Needs angular scanning setup, less portable, sensitive to vibrations	55
Chemiluminescence/luminescence	Detects light emitted from enzyme-catalyzed chemiluminescence or bioluminescent reactions	Very high sensitivity, low background noise, does not require excitation source	Limited enzyme stability, single-use, reagent dependent, short-lived signal duration	56
Fluorescence resonance energy transfer (FRET)	Monitors energy transfer between two fluorophores in close proximity	Excellent for molecular interaction mapping, real-time detection, high spatial resolution	Requires dual labeling, distance dependent, expensive reagents, photobleaching of fluorophores affects performance	57
Total internal reflection fluorescence (TIRF)	Uses evanescent field to excite fluorophores near surface only	High surface specificity, low background noise, excellent for membrane or surface studies	Only detects events near surface (∼100–200 nm), requires precise optical alignment	58
Optical waveguide light mode spectroscopy (OWLS)	Measures refractive index changes at waveguide surface	Real-time, label-free, suitable for kinetic and concentration measurements	Requires waveguide integration, niche applications, costly instruments	59
Interferometry (Mach–Zehnder interferometer, biolayer interferometry)	Measures phase shifts due to biomolecular binding on surface	Real-time, label-free, highly sensitive, suitable for kinetic profiling	Sensitive to temperature fluctuations and optical drift, requires stable operating environment	60 and 61

**Piezoelectric**				
Bulk acoustic wave (BAW) (includes QCM, TSM, PQC)	Utilizes shear or longitudinal acoustic waves that propagate through the piezoelectric substrate; binding of biomolecules induces a frequency shift proportional to mass	High sensitivity to mass changes, real-time and label-free detection, suitable for biochemical liquid samples	Fragile at high frequency, sensitive to viscosity and temperature, requires surface functionalization	62, 63 and 43
	QCM: measures changes in resonance frequency on crystal surface			
	TSM: detects changes through shear vibration, suited for liquids			
	PQC: variant of QCM using AT-cut crystals, often interchangeable in biosensing. Used interchangeably with QCM in biosensing literature due to shared operational principles			
Surface acoustic wave (SAW)	Surface-propagated acoustic waves interact with biomolecules on the sensor surface, changes in wave velocity/attenuation indicate binding	Extremely sensitive to surface interactions, fast response time, ideal for small molecule/pathogen detection, label-free	Sensitive to ambient temperature and humidity, complex and costly fabrication, limited robustness	62

## 3. Applications of point-of-care biosensors

### 3.1. Point-of-care electrochemical biosensors for disease diagnosis

Point-of-care (POC) electrochemical biosensors represent a rapidly developing and promising approach for disease diagnosis, offering the potential for fast, precise, and low-cost testing outside conventional laboratory methods.^64^ In this work, we focus primarily on its applications in diagnosing infectious diseases. Furthermore, their roles in diagnosing different infectious diseases are discussed. In Table 2, we discuss about different subtypes of electrochemical biosensors and there features.

**Table 2:** Electrochemical biosensors used in infectious disease detection, showing target biomarkers, sensing techniques, and detection limits across various diseases

Disease	Target biomarker	Biosensor type/technique	Key features/detection limit	References
Sepsis	TNF-α, IL-6, miR-155; 16S rRNA gene fragments	Electrochemical biosensor; READ device; electrochemical genosensor	Early detection in LPS-induced murine models; portable READ device for inflammation biomarkers; specific bacterial detection	64 and 16
SARS-CoV-2 (COVID-19)	Spike protein, NP, antigens; spike-ACE2 complex	Electrochemical biosensor; immunosensor; READ platform; LSG/nanomaterial-based sensors	High sensitivity (96.04%) and specificity (87.75%); applicable to diverse clinical specimens	65, 66, 13, 16 and 67
Hepatitis C virus (HCV)	E2 envelope protein; viral RNA; HCV-specific antibodies	Molecularly imprinted polymer (MIP) sensor; nucleic acid-based biosensors	Effective PoC testing	15
Malaria	Plasmodium falciparum lactate dehydrogenase (PfLDH); parasite markers (*e.g.*, cryptosporidium, trypanosoma)	Magnetoimmunoassay with magnetic beads and paper microfluidic electrodes; aptamer-based biosensors	Rapid, quantitative whole blood diagnosis; aptamer specificity for parasite detection	68 and 12
Melioidosis	Capsular polysaccharide (CPS) of *Burkholderia pseudomallei*	Electrochemical immunosensor/eELISA; READ sensor	High CPS sensitivity in serum and urine; early onsite predictability	16
Human papillomavirus (HPV)	HPV-16 DNA	Super sandwich structured biosensor with gold electrodes; nucleic acid-based electrochemical platforms	Effective interference resistance in serum; early high-risk HPV detection	2 and 15
Human immunodeficiency virus (HIV)	HIV DNA	Flexible paper-based electrochemical sensor	Highlights PoC electrochemical detection	68
General bacterial & viral infections	16S rRNA; pathogens in wastewater	Carbon-stabilized porous silicon biosensor; electrochemical genosensor	Used for UTIs, bacteremia, and pathogen monitoring in wastewater	69 and 15

#### 3.1.1. Detection of sepsis

Electrochemical biosensors have shown promise for the early detection of sepsis by monitoring biomarkers such as TNF-α, IL-6, and miR-155 in LPS-induced murine models.^64^ A rapid electroanalytical device (READ) sensor, which combines a single-use chip with a portable reader, has been used to differentiate septic from nonseptic samples *via* inflammatory biomarker detection.^16^ Electrochemical genosensors targeting specific 16S rRNA gene fragments are also being explored for rapid and precise diagnosis of bacterial infections causing sepsis.^64^

#### 3.1.2. Detection of SARS-CoV-2 (COVID-19)

Electrochemical biosensors targeting SARS-CoV-2 components such as the spike protein, NP, and antigens, or those based on spike-ACE2 interactions, have been reported for clinical samples such as blood, serum, tracheal aspirates, and nasopharyngeal swabs.^13,65,66^ An immunosensor for the spike-ACE2 complex showed excellent sensitivity (96.04%) and specificity (87.75%).^66^ The READ platform allows for COVID-19 biomarker detection and disease severity stratification.^16^ Moreover, laser-scribed graphene (LSG) and nanomaterial-based electrochemical biosensors are also important for POC diagnosis of COVID-19.^66,67^

#### 3.1.3. Detection of hepatitis C virus (HCV)

Electrochemical biosensors are crucial for early HCV diagnosis. A sensor using molecularly imprinted polymers (MIPs) targets the E2 envelope protein for effective point-of-care testing. These biosensors detect either viral RNA or HCV-specific antibodies, and nucleic acid-based platforms are also used for detecting HCV.^15^

#### 3.1.4. Detection of malaria

An electrochemical POC device for malaria detection of *Plasmodium falciparum* lactate dehydrogenase (PfLDH) from the whole blood of malaria patients has been developed. These devices show rapid detection abilities and can be used to quantitatively diagnose malaria infection. They used a magnetoimmunoassay with magnetic beads and paper microfluidic electrodes.^68^ For detecting parasites such as *Plasmodium*, *Cryptosporidium oocysts*, *and Trypanosoma* sensors are being developed. These sensors utilize specific binding molecules called aptamers. Aptamers can recognize their target and bind particularly to it. Hence, the sensors under development are aptamer-based electrochemical sensors.^12^

#### 3.1.5. Detection of melioidosis

Electrochemical immunosensors for detecting and diagnosing melioidosis are being developed. These immunosensors detect and quantify biomarkers for melioidosis. This disease can be diagnosed by the presence of a particular biomarker called capsular polysaccharide (CPS), which is found in *Burkholderia pseudomallei.* This electroanalytical immunosensor device is also known as a rapid analyzer device (READ) sensor. It has an improved ability to detect CPS in serum or urine samples and promises to predict disease in the early stage.^16^

#### 3.1.6. Detection of human papillomavirus (HPV)

Electrochemical biosensors have been developed for the detection of HPV. These sensors confirm the detection of high-risk HPV-16 DNA. The sensors are produced *via* a supersilwich structure with modified gold electrodes. When tested with human serum samples, the sensors showed strong interference resistance. Therefore, these sensors are valuable clinical diagnostic tools.^2^ Sensors are also produced on the basis of nucleic acids.^15^

#### 3.1.7. Detection of human immunodeficiency virus (HIV)

Electrochemical biosensors are used for the detection of HIV. Sensors have been developed by using flexible paper-based electrodes. Research shows that the use of electrochemical POC biosensors for detecting HIV DNA and diagnosing HIV is highly beneficial.^68^

#### 3.1.8. Detection of general bacterial and viral infections

**Fig. 5:**
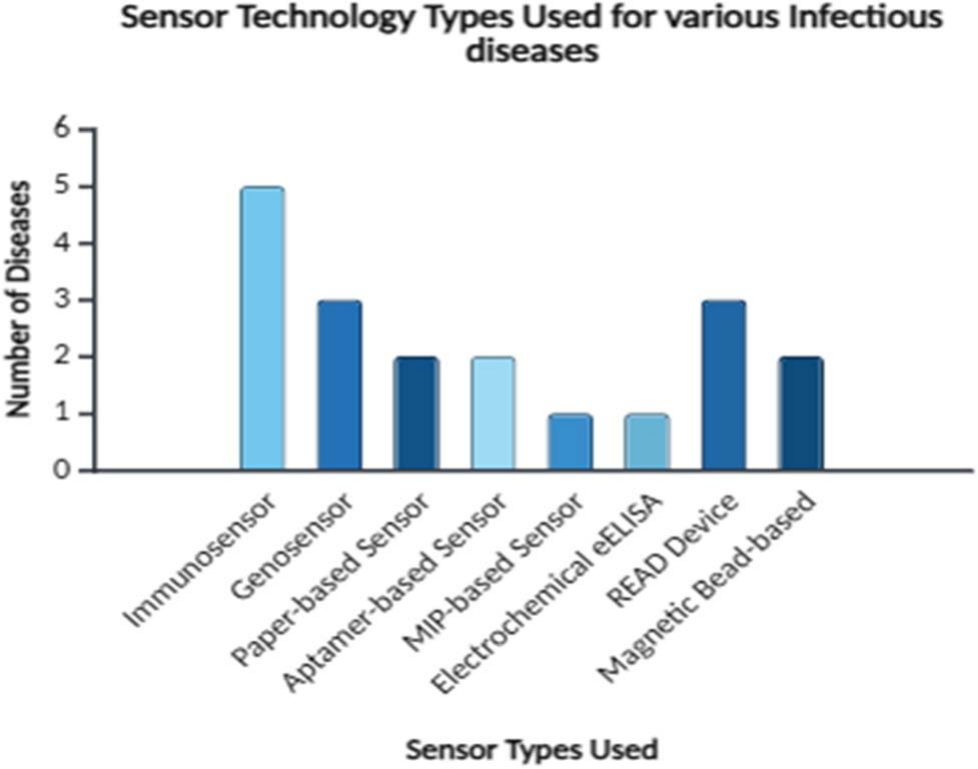
Sensor technology types used for various infectious diseases, created *via* BioRender.

Electrochemical biosensors consisting of carbon-stabilized porous silicon sensors are used for the detection of 16S rRNA for the diagnosis of infections such as urinary tract infections and bacteremia. Electrochemical genosensors are being developed. They also use 16S rRNA as a biomarker.^69^ The number of infectious diseases that spread from wastewater is increasing because of the bacteria and viruses present in them. Biosensors for detecting and diagnosing these infections are also under development (Fig. 5).^15^

Electrochemical biosensors have shown great efficiency for detecting specific biomarkers in biological samples. These methods can be used for the detection and diagnosis of many diseases. However, more changes and improvements can be made to increase the standard of biosensors and improve their performance with real-life biological samples.

### 3.2. Point-of-care optical biosensors for disease diagnosis

Since traditional diagnostic methods can be slow and occasionally inaccurate, often requiring complex equipment and trained personnel, optical biosensors offer a faster, more sensitive, and user-friendly alternative for detecting infectious diseases, especially in point-of-care settings and for widespread screening during disease outbreaks. In Table 3, we discuss about different subtypes of optical biosensors and there features.

**Table 3:** Optical biosensors used in infectious disease detection, showing target biomarkers, sensing techniques, and detection limits across various diseases

Disease	Target biomarker	Biosensor type/technique	Key features/detection limit	References
COVID-19	Anti-SARS-CoV-2 antibodies, RBD antigen, viral RNA	Fluorescence, SPR, SERS, colorimetry, photoluminescence	Low-cost, fast, visual detection, femtomolar-level sensitivity	70 and 71
HIV-1	gp120 protein, HIV-1 DNA (preantibody stage)	SPR, photonic crystal hydrogel sensor	48 fM (SPR), 4 viral particles per mL (hydrogel)	72 and 73
Hepatitis B	HBV DNA, HBsAg, HBeAg	Lateral flow (colorimetry), graphene–gold hybrid, chemiluminescent optical fiber	0.3–8.5 pM (DNA/HBsAg), 50 pg mL^−1^ (HBsAg), 0.01 fg mL^−1^ (HBeAg)	74, 75 and 76
Tuberculosis	TB antigens in sputum, nucleic acids	SPR, OLED-based sandwich hybridization, Raman spectroscopy	63 pg mL^−1^, rapid detection from processed sputum	77, 78 and 79
Malaria	PfGDH, PfLDH, infected RBC refractive index	SPR, antibody-aptamer plasmonic sensor, smartphone-based fiber-optic aptasensor	<30 fM (PfLDH), 264 pM (PfGDH), visual detection in <1 h	4, 81, 82 and 83
Ebola virus	Soluble glycoprotein (sGP), VP40, glycoproteins	Optofluidic nanoplasmonic, nanoantenna, SOI nanowire sensor	220 fg mL^−1^ (sGP), response in <5 min, real-time detection	84, 85 and 86
*E. coli*	Whole bacterial cells	Interferometric reflectance imaging (SP-IRIS)	Single-cell level, label-free, works in unprocessed samples	87

#### 3.2.1. Detection of SARS-CoV-2 (COVID-19)

Optical biosensors play an essential role in detecting SARS-CoV-2 biomarkers. A biosensor was made using the semiconductor copolymer F8T2 as a signal transducer with the sensor surface functionalized with the receptor-binding domain (RBD) of the virus spike protein. When anti-RBD antibodies bind to the antigen, a shift in the photoluminescence spectrum of F8T2 is observed. The ratio of peak intensities helps distinguish positive samples from negative samples.^70^ Innovations such as fluorescence, SPR, surface-enhanced Raman scattering (SERS) and colorimetry offer quicker and more convenient alternatives to RT–PCR, making them useful for POC testing.^71^

#### 3.2.2. Detection of human immunodeficiency virus (HIV)

The biosensor uses a DNA tetrahedron structure combined with SPR and strand displacement reactions to detect HIV-1 with high sensitivity (48 fM) without amplification, although clinical validation is pending.^72^ Another approach is a ssDNA-aptamer-linked photonic crystal (APC) hydrogel sensor used for HIV detection, which is composed of photonic crystals (PCs) made of polystyrene nanoparticles embedded in a polyacrylamide hydrogel that target the HIV gp120 glycoproteins on the virus surface, producing a visible color change when bound. It offers a detection limit of 7.1 ng mL^−1^ for gp120 and 4 viral particles per mL, with results available in just 5 minutes.^73^

#### 3.2.3. Detection of hepatitis B

A lateral flow biosensor using Au@Pt nanorods leverages their oxidase-like activity for simple colorimetric detection of HBV DNA with an 8.5 pM limit, eliminating the need for complex reagents such as hydrogen peroxide.^74^ A graphene–gold hybrid biosensor detects HBsAg in real-time with a 50 pg mL^−1^ detection limit.^75^ Another optical fiber-based immunosensor uses chemiluminescence to detect HBsAg and HBeAg at ultralow concentrations—down to 0.3 fg mL^−1^ and 0.01 fg mL^−1^, respectively—and has strong potential for early-stage diagnosis.^76^

#### 3.2.4. Detection of tuberculosis

TB diagnosis in HIV-positive patients is difficult and requires accessible tools. A portable SPR system employs gold surfaces modified with carboxylated polysaccharides and allows direct *M. tuberculosis* secretory protein (Ag85) detection from patient samples (even sputum samples) in low-resource settings.^77^ Organic light-emitting diode-based OLED-based platforms using magnetic beads and sandwich hybridization detect concentrations as low as 63 pg mL^−1^.^78^ Raman spectroscopy-based sensors offer noninvasive detection from cell-free sputum, identifying patients on TB medication.^79^

#### 3.2.5. Detection of malaria

SPR biosensors using aptamers have shown high sensitivity for malaria biomarkers such as PfGDH and PfLDH, with subpicomolar detection (∼0.77–0.84 pM) and results in under one hour of detection.^5,80^ An advanced antibody–aptamer plasmonic biosensor utilizing a gold nanoparticle array detects PfLDH in whole blood with <30 fM sensitivity, requiring no sample prep and is ideal for POC testing.^81^ A novel ohm-shaped SPR device composed of silicon oxide with tungsten as a cavity resonator tracks refractive index shifts in infected red blood cells to differentiate infection stages.^82^ Another example shows a portable, smartphone-based fiber-optic aptasensor that targets PfGDH, with a 264 pM detection limit and suitability for low-resource use.^83^

#### 3.2.6. Detection of Ebola virus

One approach for detection uses a 3D plasmonic nanoantenna array-based biosensor that can detect sGP at 220 fg mL^−1^, which is 240 000 times more sensitive than the methods used in current tests.^84^ A nanowire silicon-on-insulator (SOI) biosensor that senses Ebola virus VP40 immune complexes in 200–300 seconds per test sample was used.^85^ Another method uses a label-free optofluidic nanoplasmonic sensor that was developed for Ebola virus detection using VSV-pseudotyped Ebola (PT-Ebola) as a model. It consists of a biosensor surface functionalized with antibodies against Ebola glycoproteins that help detect virus binding by measuring a 14 nm redshift in resonance.^86^

#### 3.2.7. Detection of *E. coli*

The SP-IRIS system detects *E. coli* by capturing individual bacteria on an antibody-coated sensor surface. When the sample is applied on the surface, *E. coli* binds to the antibodies, which are then visualized as tiny dark spots. It uses interferometric reflectance imaging; hence, no fluorescent labels are needed. This method is helpful in identifying single bacteria and their size/shape, allowing quick and accurate detection in complex samples.^87^

The application of optical biosensors has revealed their ability to detect viral and bacterial infections with high sensitivity and rapid response. The use of diverse optical techniques allows flexibility. Nonetheless, improving their performance in minimally processed samples is essential for real-world diagnostics.

### 3.3. Point-of-care piezoelectric biosensors for disease diagnosis

While optical biosensors are known for their great sensitivity and real-time monitoring ability, piezoelectric biosensors are unique to the table; they can detect mass changes without labels. This makes them a powerful tool for diagnosing infectious diseases. They are fast, accurate, and much more straightforward than traditional methods, which often involve lengthy procedures, complex steps, and high costs. Studies have shown that these biosensors can effectively detect a range of viral and bacterial infections, whether sudden or chronic. In Table 4, we discuss about different subtypes of piezoelectric biosensors and there features.

**Table 4:** Piezoelectric biosensors used in infectious disease detection, showing target biomarkers, sensing techniques, and detection limits across various diseases

Disease	Target biomarker	Biosensor = type/technique	Key features/detection limit	References
Hepatitis B	HBV DNA	QCM with DNA probe, piezoelectric actuator with CRISPR-RPA system	Femtomolar-level sensitivity; reusable up to 5×, amplification-free detection *via* CRISPR integration	89, 90 and 91
Tularemia	Anti-Francisella antibodies	Gold electrode-based piezoelectricimmunosensor	Detects antibodies in <10 minutes	91
*Salmonella* spp.	Anti-Salmonella antibodies	QCM with antibody immobilization, proteus-adaptable piezoelectric sensors	Frequency shift sensitivity down to 20 Hz; 10 CFU mL^−1^ achieved within 3 hours	91 and 92
HIV	HIV-1 and HIV-2 antigens	Surface acoustic wave (SAW) piezoelectric biosensor	Detects 12 TCID_50_ for HIV-1 and 87 TCID_50_ for HIV-2; works in serum; portable PoC design	93 and 91
Dengue	NS1 antigen	QCM with bacterial cellulose nanocrystals,MEMS-based cantilever biosensor	Detection limitof 0.1 μg mL^−1^; rapid, label-freemulti-disease detection capability	94, 95 and 96
Tuberculosis	IS6110 gene, TB antigens, VOCs	QCM with DNA probe, gold nanoparticles, SPQCfor VOC detection	Detection: 30 CFU mL^−1^, sputum-compatible; VOC-based TB screening	95, 97 and 98
COVID-19	Spike protein antigens, respiratory rate	PVDF-based piezoelectric cantilever, mattress-integrated sensor,FFT and PSD signal analysis	Micro/nanogram detection; 75% sensitivity and 83% NPV for SIRS; point-of-care respiratory monitoring platform	99 and 100

#### 3.3.1. Detection of hepatitis B virus (HBV)

A piezoelectric biosensor utilizing a 9 MHz quartz crystal microbalance (QCM) with a gold-coated electrode was developed to detect HBV DNA *via* nucleic acid hybridization. The immobilization of the probe *via* polyethyleneimine–glutaraldehyde (PEI-Glu) crosslinking enhanced the stability and sensitivity, enabling detection in the range of 0.02–0.14 μg mL^−1^, with successful reuse of the probe up to five times without a loss in performance.^88^ A QCM-based platform by Giamblanco *et al.* employed immobilized ssDNA probes for label-free detection of long HBV DNA fragments with femtomolar sensitivity, thereby avoiding amplification.^89^ Recently, piezoelectric actuators were integrated with a digital RPA-CRISPR/Cas12a assay for droplet fusion, enabling rapid and quantitative detection of HBV nucleic acids.^90^

#### 3.3.2. Detection of *Francisella tularensis* (tularemia)

A piezoelectric immunosensor with a gold-coated electrode was developed for the rapid detection of antibodies against *Francisella tularensis*. Antigens from mice infected with tularemia were immobilized on the sensor surface, enabling antibody identification within 10 minutes. This approach significantly outperforms conventional dot blot assays, which typically identify tularemia only in later stages, highlighting the sensor's efficacy for early-stage diagnosis.^91^

#### 3.3.3. Detection of *Salmonella* spp

A quartz crystal microbalance (QCM)-based piezoelectric biosensor coated with anti-Salmonella antibodies that exhibited high sensitivity and was capable of identifying frequency changes as minimal as 20 Hz. Compared with traditional culture techniques, this technique provides a faster and more effective detection process.^91^ Additionally, a related platform targeting *Proteus* spp. *via* 16S rRNA and signal amplification achieved a sensitivity of 10 CFU mL^−1^ within 3 hours; this methodology can also be adapted for detecting *Salmonella*, underscoring the flexibility of piezoelectric systems in bacterial pathogen detection.^92^

#### 3.3.4. Detection of human immunodeficiency virus (HIV)

Surface acoustic wave (SAW)-based piezoelectric biosensors coated with HIV-specific antibodies have enabled rapid and label-free detection of HIV-1 and HIV-2 by monitoring mass-induced phase shifts. The reported detection limits are 12 TCID_50_ for HIV-1 and 87 TCID_50_ for HIV-2, with adequate performance in human serum.^93^ These sensors are specifically designed to work in settings where resources are limited. However, over time, studies have shown that portable biosensors can become less effective after being regenerated several times. This highlights the importance of developing more durable materials and enhanced regeneration techniques.^91^

#### 3.3.5. Detection of dengue

Quartz crystal microbalance (QCM)-based piezoelectric biosensors targeting the NS1 antigen have enabled label-free and real-time dengue detection through antigen–antibody-induced frequency shifts. The use of bacterial cellulose nanocrystals significantly improved the sensitivity, reaching detection limits as low as 0.1 μg mL^−1^, and reduced the need for complex sample preparation.^94,95^ Additionally, MEMS-based piezoelectric biosensors with polysilicon cantilevers have shown potential for multiplexed vector-borne disease detection, with simulations confirming a selective response to dengue virus antigens.^96^

#### 3.3.6. Detection of *Mycobacterium tuberculosis*

P-quartz crystal microbalance (QCM)-based piezoelectric biosensors have shown high efficacy in detecting *Mycobacterium tuberculosis* by monitoring frequency shifts caused by antigen–antibody binding events.^95^ Earlier versions of these sensors used biotinylated DNA probes attached to gold electrodes targeting the IS6110 gene, enabling specific and amplification-free detection with minimal cross-reactivity. Recent approaches have integrated gold nanoparticles and enzyme-assisted signal amplification, achieving sensitivities as low as 30 CFU mL^−1^ even in clinical sputum samples.^97^ Additionally, new multichannel shear-mode piezoelectric quartz crystal (SPQC) systems have been developed to sense changes in volatile organic compounds (VOCs) and conductivity, which broadens their application for TB screening in both medical and environmental settings.^98^

#### 3.3.7. Detection of SARS-CoV-2

Piezoelectric biosensors provide a sensitive, rapid, and label-free alternative to RT–PCR for SARS-CoV-2 detection. Devices employing PVDF or 128° YX lithium niobate detect frequency shifts caused by antigen–antibody binding. Kabir *et al.* (2021) reported a comb-structured cantilever functionalized with spike proteins, enabling real-time signal validation and minimizing false positives.^99^ Another system employing PVDF microcantilevers translated piezoelectric strain into voltage signals, which were analyzed with fast Fourier transform (FFT) and power spectral density (PSD) analysis, achieving high sensitivity in the micro- and nanogram range. Additionally, Kobayashi *et al.* (2024) developed a mattress-based piezoelectric sensor system that was capable of noninvasive respiratory monitoring in COVID-19 patients.^100^ This system uses a piezoelectric film under the mattress to calculate a 40 minutes frequency distribution of the respiratory rate (M40FD-RR), with a sensitivity of 75% and a negative predictive value of 83%. These advances highlight the versatility of piezoelectric biosensors for both direct virus detection and patient monitoring.

Piezoelectric biosensors have shown effective detection across pathogens, including HBV, TB, and HIV, with rapid, label-free operation. The mass-sensitive mechanisms of these materials are advantageous. However, challenges such as limited durability, regeneration issues, and sensitivity to sample complexity need resolution for broader adoption.

## 4. Limitations in detecting diseases

Current point-of-care (POC) biosensing systems are emerging as novel alternatives to traditional methods such as viral isolation, PCR, ELISA, culture, microscopy, and mass spectrometry.^101^ While these conventional techniques have long been considered the gold standard, they often have several drawbacks. These methods take considerable time, cost more, and often require skilled personnel and sophisticated setups.^101–103^ In real-world clinical settings, the challenges include variations in sensitivity, specificity, and stability due to biological variability or interference from the sample matrix. For example, label-free detection methods often face issues such as nonspecific binding, which can distort results and lead to misinterpretation.^102^ Although technology has come a long way, integrating components such as transducers, detection, and microfluidic sample preparation into a single platform remains challenging. While point-of-care tests such as lateral flow assays offer fast and easy-to-use techniques such as PCR and ELISA, they still outperform other methods in terms of sensitivity and specificity.^104^ Bringing POC systems into real-world use also faces logistical hurdles. The devices may malfunction in tropical environments; power supply and cold storage can be unreliable or unavailable in low-income regions, and access to clinical samples is often limited. In addition to these technical barriers, practical challenges include limited trust in new tools, time constraints in clinical settings, weak patient follow-up, and inefficient referral processes.^102^

## 5. Challenges in the development of POC biosensors

Currently, the procedures used for the diagnosis of any disease or infection are limited. These include slow and tedious methods; expensive treatment, which also requires specialized and automatic instruments; the use of experienced healthcare workers; and extensive preparation for testing samples.^105^ Considerable time and technical support will be required for developing commercial POC biosensors.^17^ For the detection of analytes at low concentrations, achieving high sensitivity is crucial. This requires surface modifications and signal amplification techniques. Similarly, it is very common.^12,64^ While POC devices are designed for rapid output, some current biosensors still have long processing times.^64,69^ Additionally, as the goal of POC biosensors is to provide affordability, some highly sensitive components, such as microfluidic chips, are expensive to produce. For large-scale implementation, affordability and simple production procedures are crucial.^2^ It is challenging to preserve the stability of recognition elements such as aptamers or enzymes, particularly in real-life biological samples or extreme conditions.^4,6^ To advance the commercialization of POC biosensors, overcoming these significant issues with reliable material design and integrative systems is crucial. To assess their practical utility, the main biosensor types were compared *via* the REASSURED criteria. Table 5 outlines how electrochemical, optical, and piezoelectric platforms meet these standards.

**Table 5:** Evaluation of biosensor classes against the REASSURED criteria for point-of-care applications

Criteria	Electrochemical biosensors	Optical biosensors	Piezoelectric biosensors
Real-time connectivity	Easy integration with portable electronic devices and smartphone interfaces for real-time signal transmission and data sharing^13^	Lacks real-time connectivity although newer smartphone-based fluorescence and SPR sensors are emerging^38^	Can be integrated with MEMS or QCM setups, but less frequently used^43^
Ease of specimen collection	Ideal for minimally invasive fluids (such as blood, urine, or saliva) that require little pretreatment, particularly in disposable and paper-based formats^103^	Suitable for direct analysis of biological fluids especially in label-free SPR and interferometric formats^52^	Accepts complex fluids and is generally label-free^62^
Affordability	Installation of the device in outdoor environments is possible by low-cost production and cheap materials like screen-printed electrodes^103^	Generally, higher cost, but smartphone and paper-based platforms are lowering expenses.^82,83^	Uses costlier materials like quartz but MEMS-based formats are emerging^46,62^
Sensitivity	Nanomaterial-modified electrodes and signal amplification strategies leads to high sensitivity achieving picogram to femtomolar LOD^13^	High sensitivity, especially in SPR, LSPR and fluorescence-based platforms^38^	Sensitivity ranges from microgram to femtomolar depending on the system^89,90^
Specificity	Aptamers, enzymes, or antibodies that are specific to target analyte leads to high specificity^13^	High specificity *via* surface-bound antibodies, aptamers, or probes with controlled functionalization^20^	Uses antibody or DNA probes for good selectivity^88,89^
User friendliness	Understandable signal output using portable or smartphone-integrated readers, often aiming for “sample-in-answer-out” functionality, requires minimal training^68^	Often need complex optical setups, but recent advancements can simplify its operation^20^	Often requires calibration but can be designed for simple use^43^
Rapid and robust	Provides rapid outputs often within minutes and performance is stable under varying operating conditions^68^	Detection times typically range from 5–30 minutes^84^	Provides results in under 10 minutes and works well in harsh settings^62^
Equipment-free	Often works without large instruments, especially in outdoor environments^13^	Often requires optical detectors, but lateral flow and fiber-based platforms reduce equipment needs^20^	Often needs frequency counters but MEMS designs reduce this requirement^46^
Deliverable to end users	Highly deliverable for *in situ* diagnosis, mass screening and general health monitoring directly at the point-of-care^13^	New portable and smartphone-based optical sensors are easier to use, but older systems stay limited to labs^82,83^	Shows potential for wearables and field use but needs further development^43^

## 6. Advances in POC biosensors

Recent progress in materials science, nanotechnology, and device design has significantly improved point-of-care biosensors, making them more sensitive, faster, and easier to use for detecting infectious diseases. Electrochemical biosensors are central because they are simple, cost-effective, and provide quick results. Nanomaterials such as nanoparticles, nanostructures, and metal–organic frameworks (MOFs) improve sensitivity and reliability.^47,105^ For example, gold/copper oxide nanocubes amplify signals in SARS-CoV-2 immunosensors, achieving low detection limits.^65^ Innovations include enzyme-free sensors with Ni-Co@C nanocages for glucose monitoring,^106^ DNA origami electrochemical interfaces for specific viral detection,^107^ and integration with glucometers *via* split enzyme technology.^69^ There are several issues, even though nanomaterials such as MOFs and nanocages improve the sensitivity of POC biosensors. Although issues such as uniform synthesis and growth continue, flexible Ni-MOF-based electrodes, for example, show stable biosensor performance. MOF or nanocrystal-coated QCM surfaces increase the specificity of piezoelectric sensors, but they also need to be carefully functionalized.^43,98^ Optical biosensors have evolved with smartphone-coupled paper platforms for portable colorimetric analysis,^108^ biomimetic nanopillar sensors for label-free influenza A detection,^109^ and biodegradable lotus root fiber waveguides for fluorescence detection in resource-limited settings.^110^ Topologically integrated photonic circuits provide robust multiplexed biomarker detection.^111^ Compact platforms such as photonic chips and SERS substrates allow multiplexed optical biosensors to detect multiple biomarkers, including proteins, small molecules, and miRNAs, simultaneously offering high sensitivity and real-time analysis, making them appropriate for infectious disease panels in low-resource environments.^112^ Reliable multianalyte detection in dynamic or decentralized environments is made possible by the use of an FFT and AI-based filtering in piezoelectric systems to reduce signal drift under unstable conditions.^97^ All three biosensing platforms have successfully integrated CRISPR/Cas systems. These systems enhance specificity and signal amplification and achieve ultralow (femtomolar) LODs in electrochemical biosensors.^101^ Optical biosensors, which are generally highly sensitive but limited by recent and equipment limitations, use CRISPR/Cas12a combined with quantum dot-linked DNA probes to generate a fluorescence signal upon target recognition.^60^ For accurate and quick HBV detection, piezoelectric biosensors have used CRISPR *via* piezoactuated droplet fusion in digital RPA-Cas12a platforms.^90^ Piezoelectric biosensing advances include the use of QCM-D for real-time monitoring of bacterial lysis and assessment of antimicrobial effects without labels.^43^ By increasing signal clarity and recognition accuracy, machine learning (ML) improves biosensor performance; for example, ML aids in optimizing biomolecular interaction patterns for improved selectivity in synthetic polymer-based and molecularly imprinted sensors.^64^ In optical biosensors, dual-core gold-coated PCF-SPR systems use structured fibers and light–plasmon coupling for label-free detection of refractive index changes (1.31–1.40 RI) over 400–900 nm. Machine learning algorithms such as the random forest regressor improve detection clarity, reduce noise, and support real–time analysis. These advances collectively move toward portable, accurate, low-cost diagnostic tools for infectious diseases, especially in resource-limited environments.

## 7. Future prospects

Future efforts aim to increase the accuracy, stability, and field usability of point-of-care (POC) biosensors. Advancements in bioconjugation strategies are expected to significantly improve sensitivity and specificity. Ongoing research is focused on overcoming practical limitations in electrochemical biosensors, particularly for pathogenic bacteria detection, such as lowering detection thresholds, shortening assay times, enabling analysis of unprocessed complex samples, and distinguishing specific pathogens in polymicrobial environments.^103^ Innovative molecular strategies such as the amplicon binding split trehalase assay (ABSTA) are being explored as next-generation diagnostic tools for gene-specific pathogen detection, although further refinement is needed for routine deployment.^69^ Additionally, addressing antibiotic resistance remains a critical frontier, driving the demand for platforms capable of rapid resistance profiling at the point of care.^103^ The development of generalized integrated systems capable of analyzing multiple types of clinical samples (*e.g.*, urine, blood, saliva) for diverse pathogens could maximize the practical impact of POC diagnostics. Standardization challenges in nanomaterial integration and electrode surface chemistry affect reproducibility and regulatory approval in electrochemical biosensors. Optical biosensors face difficulties owing to variability in surface modification processes (such as thiol–gold SAM formation and silanization), batch-to-batch differences in nanoparticle synthesis and reliance on expensive components such as spectrometers and lasers, which limit scalability. Piezoelectric biosensors show promise through MEMS-based fabrication and flexible PVDF composites,^99^ but consistent calibration and reproducibility in field settings remain key regulatory hurdles.^97^ With the continued evolution of computational tools, automated systems, and miniaturized devices, the transition from laboratory prototypes to real-world diagnostic applications is likely to accelerate.^113^

## 8. Conclusion

Recent breakthroughs in materials science, nanotechnology, and device engineering have greatly advanced POC biosensors, improving sensitivity and user-friendliness and reducing detection time. Electrochemical biosensor advancements include the use of widely used nanomaterials (nanoparticles, nanostructures, and MOFs) to improve signal strength and sensitivity. For targeted detection, enzyme-free sensors and DNA origami-based interfaces are becoming more popular. Commercial glucometer compatibility is made possible by the integration of DNA-binding proteins with split enzyme technology. Optical biosensor advancements include smartphone-coupled paper-based platforms for portable analysis. Label-free detection of viral antigens *via* reflectance measurements is made possible *via* biomimetic nanopillar-based sensors. Eco-friendly optical waveguides provide biodegradable, affordable options for fluorescence-based pathogen detection. Robust, miniaturized multiplexing platforms are offered by topologically integrated photonic circuits. For high-precision nucleic acid detection, the CRISPR/Cas system is being employed increasingly frequently. Piezoelectric biosensor advancements include the use of QCM-D to assess bacterial lytic activity in real-time, offering a label-free method for evaluating antimicrobial efficacy. The performance, portability, and accessibility of electrochemical, optical, and piezoelectric biosensors are all significantly emerging due to technological advancements, especially the use of nanomaterials and their integration with digital devices such as smartphones. POC biosensors show great promise for rapid and affordable infectious disease diagnosis, but challenges remain in enhancing performance, bridging research with clinical applications, and addressing regulatory and design considerations for effective use.

## Author contributions

Mansi Parihar, Niharika WN, Sahana: data curation, investigation, visualization, methodology, writing—original draft. Rajib Biswas: review & editing. Budheswar Dehury, Nirmal Mazumder: conceptualization; supervision; acquisition of funding, review & editing.

## Conflicts of interest

The authors declare no conflicts of interest.

## Data Availability

No primary research results, software or code have been included and no new data were generated or analysed as part of this review.
